# Efficacy and safety of magnetic resonance imaging- vs. computed tomography-guided reperfusion in acute ischemic stroke: a systematic review and meta-analysis

**DOI:** 10.3389/fneur.2026.1862054

**Published:** 2026-08-03

**Authors:** Sabia Afreen, Michelle Sámano Sánchez, Montserrat Villa Sánchez, Angelo Magallanes Bajana, Ajay Kumar, Ricardo A. Sotelo Ortega, Ruth Diaz Revelo, Edelvira Cerdan Abad, Beatriz González de Montecinos, Stephani Carolina Salvatierra Moreno, Victor Sebastian Arruarana, Ernesto Calderon Martinez

**Affiliations:** 1 Shanghai Medical College, Fudan University, Shanghai, China; 2 Facultad de Medicina, Universidad Autónoma de Sinaloa, Culiacán, México; 3 Centro de Estudios Universitarios Xochicalco, Tijuana, México; 4 Universidad de Guayaquil, Guayaquil, Ecuador; 5 Isra University Faculty of Medicine and Allied Medical Sciences, Hyderabad, Sindh, Pakistan; 6 Universidad Autónoma de Baja California, Facultad de Medicina, Mexicali, México; 7 Universidad Católica de Honduras, San Pedro Sula, Honduras; 8 Our Lady of Fatima University College of Medicine, Valenzuela, Philippines; 9 UVM Universidad del Valle de México, Facultad de Medicina, Reynosa, Tamaulipas, México; 10 Universidad de Los Andes, Mérida, Venezuela; 11 Department of Internal Medicine, Brookdale University Hospital and Medical Center, Brooklyn, NY, United States; 12 Department of Internal Medicine – Transmountain, Texas Tech University Health Sciences Center El Paso, El Paso, TX, United States

**Keywords:** acute ischemic stroke, computed tomgraphy (CT), intravenous thrombolysis, magnetic resonace imaging (MRI), mechanical thrombectomy

## Abstract

**Objective:**

Acute ischemic stroke (AIS) is a leading cause of death and disability. Timely reperfusion improves outcomes, but optimal patient selection depends on neuroimaging; CT is fast and widely available, whereas MRI offers greater precision in identifying salvageable brain tissue. This systematic review (SR) and meta-analysis evaluates the comparative effectiveness of MRI-guided reperfusion (MRI-R) vs. CT-guided reperfusion (CT-R) in adult AIS.

**Methods:**

We conducted a PRISMA-compliant SR and searched PubMed, Embase, Cochrane Library, Web of Science, CINAHL and Google Scholar for English or Spanish studies comparing MRI-R vs. CT-R in adult AIS. Randomized trials, cohort, and case-control designs were included. Primary outcome was mortality; secondary outcomes were 90-day functional independence modified Rankin Scale (mRS) 0–2) and symptomatic intracranial hemorrhage (sICH). Data were pooled with a random-effects model; risk of bias was assessed using Cochrane RoB 2.0 and ROBINS-I tools.

**Results:**

From 11,578 records, 13 studies (27,313 patients) met inclusion criteria. Meta-analysis of 11 studies (27, 197 patients) showed MRI-R was associated with increased 90-day mRS ≤2 (RR 1.28, 95 % CI 1.06–1.55; *p* = 0.01; I^2^ = 82.6 %), reduced mortality (RR 0.72, 95 % CI 0.62–0.84; *p* = 0.002; I^2^ = 0 %) and lowered sICH (RR 0.59, 95 % CI 0.45–0.78; *p* = 0.002; I^2^ = 7.6 %) compared with CT-R. Subgroup analyses by CT comparator type (NCCT-only or multimodal CT) and reperfusion strategy (IVT, MT, or both) for successful reperfusion and 90-day functional independence showed no statistically significant differences between groups.

**Conclusion:**

MRI-R was associated with improved functional outcomes, reduced mortality and lower sICH rates than CT-guided strategies. However, these findings should not be interpreted as definitive superiority given the high heterogeneity in functional outcomes, baseline patient characteristic differences between imaging groups, and detected publication bias. CT remains essential for rapid hyperacute assessment, while in well-equipped centers, MRI can improve patient selection through detailed tissue characterization, provided this does not delay reperfusion and patients have no contraindications. Further, prospective, randomized studies are needed to confirm these findings.

**Systemic review registration:**

https://www.crd.york.ac.uk/PROSPERO/view/CRD420251165578, CRD420251165578

## 1 Introduction

Acute ischemic stroke (AIS) is one of the most significant causes of death and long-term disability worldwide, accounting for nearly 85% of all stroke cases (1). Reperfusion therapy advancements like intravenous thrombolysis (IVT) and mechanical thrombectomy (MT) have significantly improved patient outcomes when given within the appropriate therapeutic windows (2). Neuroimaging is essential in helping clinicians recognize the infarct region, assessing tissue viability and determining the extent of injury (3).

Non-contrast CT (NCCT) remains the first-line modality in most emergency departments since it is rapid, widely available, and inexpensive. However, it has limited sensitivity for early ischemic changes and cannot reliably distinguish the non-viable infarct core from possibly salvageable penumbral tissue (4, 5). MRI, especially diffusion-weighted imaging (DWI) and perfusion-weighted imaging (PWI), provides better accuracy in detecting early ischemia, defining the infarct core and overall ischemic region, while also providing a broader range of information than regular CT (6).

Increasing interest in MRI-guided reperfusion strategies has led to studies comparing it with CT-based selection with mixed results. Some studies report similar clinical outcomes, whereas others suggest MRI to be superior in identifying candidates likely to benefit from thrombolytic treatment (7, 8). Higher costs, limited availability, and safety concerns in critically ill patients continue to pose practical challenges MRI using it for acute stroke care (9).

This systematic review (SR) aims to determine whether MRI-based reperfusion (MRI-R) strategies offer better mortality and functional outcomes than CT-based reperfusion (CT-R) in adults with AIS, to help refine imaging protocols and support evidence-based stroke management.

## 2 Materials and methods

### 2.1 Methodology

The present SR followed the recommendations and criteria established by the Preferred Reporting Items for Systematic Reviews and Meta-analyses (PRISMA) reporting guidelines (10, 11) The protocol was pre-registered at the International Prospective Register of Systematic Review (PROSPERO) with the identifier code CRD420251165578 (12).

### 2.2 . Eligibility criteria

#### 2.2.1 . Types of study

This SR included studies published from the inception to October 9, 2025, limited to English and Spanish. Eligible study designs will include only randomized controlled trials (RCTs), case-control, cohort, and cross-over studies. Studies were excluded if they lacked sufficient methodological detail, present duplicate data, or failed to provide information after attempts to contact the original authors.

#### 2.2.2 . Types of participants

Eligible participants will include adults (≥18 years) who meet the diagnostic criteria for AIS as defined by the original study authors. Studies including participants with other stroke types (e.g., hemorrhagic, lacunar, or unspecified etiologies) will be excluded. Studies reporting exclusively on extended-window AIS populations were excluded.

#### 2.2.3 . Types of interventions/exposures and comparators

This review evaluates the effects of MRI-R therapy; either IVT or MT, guided by DWI and PWI in patients with AIS. Comparator interventions will include reperfusion therapy guided by NCCT imaging within the predefined study population. Studies with insufficiently reported interventions, exposures or comparators will be excluded.

#### 2.2.4 . Outcomes

The primary outcome of this review is mortality. Secondary outcomes include functional outcomes as measured by the mRS and symptomatic intracranial hemorrhage (SICH). Studies will be excluded if they do not report data on at least one of these predefined outcomes.

### 2.3 Searching methods

A systematic search was initially conducted on October 10th 2025 on Pubmed/MEDLINE, Cochrane, Web of Science, EMBASE, CINDAHL and Google Scholar using the following terms: “stroke”, “brain ischemia”, “cerebral infarction”, “magnetic resonance imaging”, “diffusion weighted imaging”, “perfusion MRI”, “computed tomography”, “CT-guided therapy”, “CT perfusion”, “thrombolysis”, “thrombectomy”, “reperfusion therapy”. All detailed search strategies can be found in the supplementary material (Supplementary Tables 1–6).

### 2.4 . Selection of studies

All references were exported to Rayyan (Rayyan Systems Inc., Cambridge, MA, USA). (13) Duplicates were removed. Two authors independently completed the eligibility assessment, first by title and abstract analysis and, subsequently, full-text assessment was independently reviewed by two authors. In disagreements between reviewers, a third reviewer helped reach a consensus.

### 2.5 . Data extraction

Three independent reviewers extracted the data, and disagreements were resolved by consensus. When multiple overlapping reports from the same study were identified, the information from the one containing the most relevant information or the first published report was included. Extracted data included sample sizes, intervention types, and measured outcomes. Conventional methods were used for data extraction, complemented by specialized tools like WebPlotDigitizer (Automeris, Austin, TX, USA) for digitizing data from graphs, Cochrane Calculator for statistical conversions, and StatsToDo for advanced calculations (14–16). Additional extracted data included subgroup characteristics like country of study, risk of bias levels, and study design.

### 2.6 . Assessment of risk of bias in included studies

Quality of included studies was assessed according to Cochrane guidelines, using Cochrane RoB 2.0 tool for RCTs and ROBINS for non-randomized studies (17, 18). Two independent reviewers evaluated the risk of bias in each study, and disagreements were resolved by a third, blinded reviewer.

### 2.7 . Statistical analysis

Meta-analysis was performed using R version 3.4.3 (R Core Team) with the meta and metafor packages (19). Pooled effects of the outcomes were examined using a random-effects meta-analysis (DerSimonian-Laird approach) (20). When insufficient number of studies were available, only a qualitative analysis of the results was performed. Effect sizes were expressed as relative risk (RR), mean difference (MD), or standardized mean difference (SMD) with a 95% CI. The I^2^ statistic assessed heterogeneity, and the following cut-off values were used for interpretation: < 25%, 25–50%, and >50% were considered small, medium, and large heterogeneity, respectively (21). For all outcomes, sensitivity analyses using the leave-one-out method were performed to determine the influence of individual studies on the overall effect (22). Egger's regression test was used to examine publication bias when 10 or more reports with the same outcome were available (23). Whenever possible, subgroup analyses were planned based on risk of bias and heterogeneity. For subgroup analyses based on CT-imaging modality, multimodal CT was defined as NCCT combined with CTA and/or CTP, whereas NCCT-only studies were analyzed separately.

## 3 Results

### 3.1 Study selection

**Figure 1:**
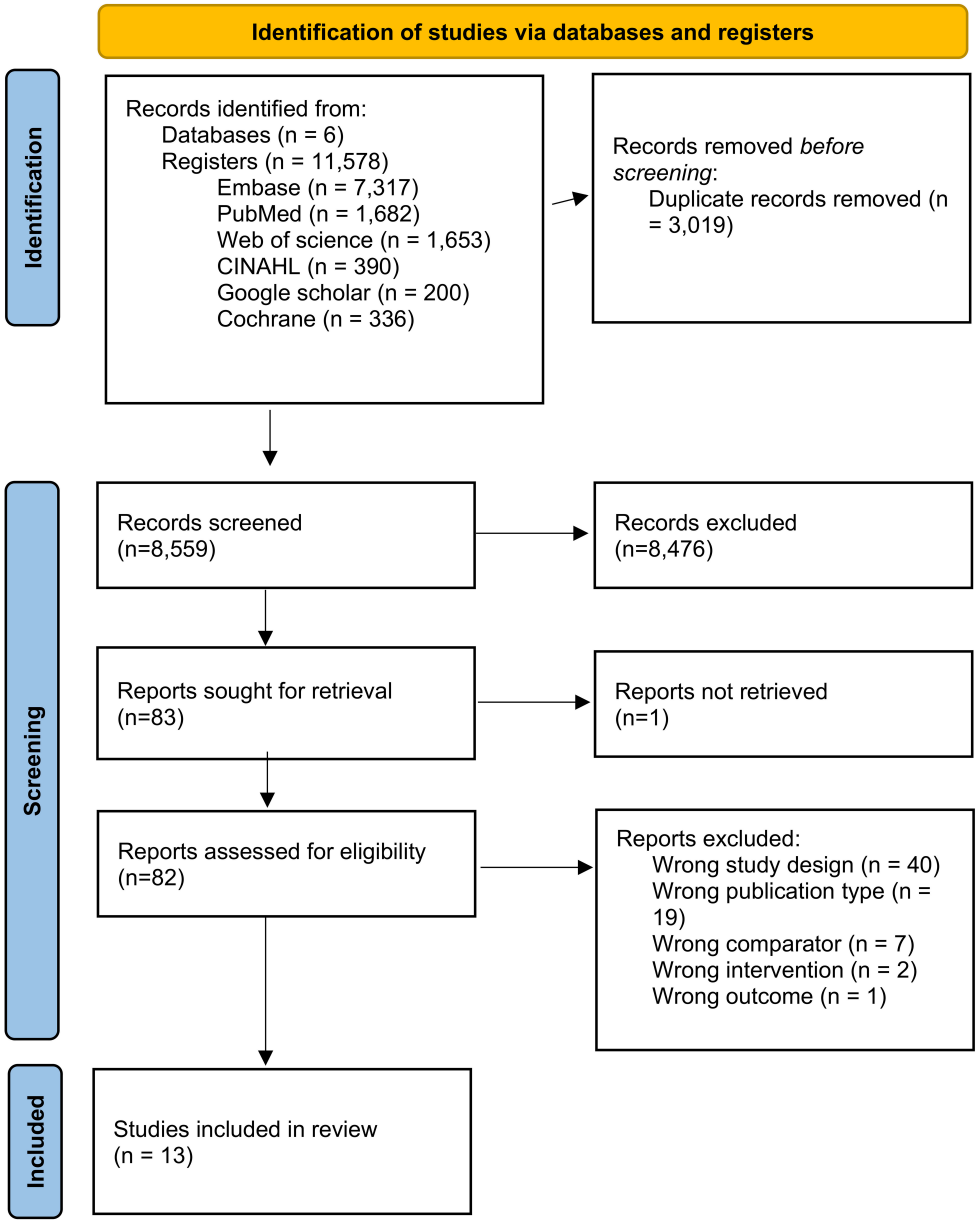
This flowchart is the PRISMA flow diagram, it outlines the systematic process of identifying and screening studies that led to the inclusion of **13** studies in this review.

We initially identified 11,578 articles across six databases. After removing 3,019 duplicates, 8,559 remained for screening, with 8,476 articles excluded. Of 83 articles identified for retrieval, 1 was excluded due to an unavailable full-text, leaving 82 for full-text eligibility assessment. Following this, 69 were excluded. Ultimately, 13 studies were included in this review. This process is illustrated in the PRISMA flow diagram (Figure 1), and the reasons for full-text article exclusion are detailed (Supplementary Table 7).

### 3.2 Characteristics of included studies

This review included 13 studies with a cumulative sample size of 27,313 individuals. The geographic distribution was diverse: the United States contributed 15.4% of the studies, Germany also accounted for 15.4%, while Denmark, Japan, Austria, China, France, and Switzerland each represented 7.7%. Around 76.9% of studies used a cohort design; both prospective and retrospective, RCTs compromised 15.4% of included studies, and quasi-randomized controlled trials (QRCTs) accounted for 7.7% of studies. In terms of study setting, 46.2% were conducted in multicenter environments, while 53.8% were single-center. All studies evaluated reperfusion therapy. Thrombolysis was assessed in 53.8% of included studies, combined thrombolysis and thrombectomy in 38.5%, and one study (7.7%) focused exclusively on thrombectomy alone. MRI served as the primary imaging modality for intervention groups in all studies, whereas CT-based imaging techniques were used for controls. The duration of follow-up varied across studies, with 76.9% reporting outcomes at 3 months by primarily assessing functional independence (mRS 0–2), while a few evaluated earlier endpoints such as mortality or safety; the remaining 15.4% assessed outcomes at 1 month, and 7.7% at seven days.

Across the included studies, baseline clinical and angiographic characteristics were largely balanced between patients evaluated with MRI and those assessed with CT. Four studies encompassing 291 participants reported baseline NIHSS scores, with patients in the MRI group showing marginally lower values, suggesting slightly less severe neurologic impairment at presentation. Six studies (*n* = 6,355; MRI = 1,324; CT = 5,031) examined internal carotid artery (ICA) occlusion, revealing similar frequencies between imaging modalities. Five studies (*n* = 1,717; MRI = 954; CT = 763) evaluating M1 occlusions and three studies (*n* = 973; MRI = 438; CT = 535) evaluating M2 occlusions demonstrated no clinically relevant differences between groups, with consistent results across cohorts. Overall, findings were consistent across studies with minimal heterogeneity, indicating that imaging modality did not substantially influence baseline severity, vascular distribution, or subsequent therapeutic decisions.

Across studies, MRI was consistently associated with longer imaging and workflow intervals, including door-to-needle (DTN), door-to-puncture (DTP), and onset-to-treatment times. However, these delays did not consistently translate into worse outcomes. Meta-analysis was not performed due to limited extractable data. Functional independence at 90 days (e.g., mRS 0–2) was similar or higher with MRI in 61.5% of studies, with several reporting statistically significant benefits. Rates of sICH were comparable or lower in MRI-based groups across 69.2% of studies, and mortality rates were similar or slightly lower in 53.8%, though statistically significance was not consistently reached. Overall, both imaging modalities demonstrated comparable safety profiles, with no significant increase in sICH or mortality associated with MRI. Differences were most noted in workflow timing, imaging precision, with modest improvements in 90-day independence favoring MRI, particularly in moderate-to-severe stroke cases. These findings are summarized in Table 1 (7, 24–35) and Supplementary Table 9.

**Table 1:** Characteristics of included studies and general outcomes.

Study	Country	Study type	Center	Case	Control	Reperfusion therapy	Total sample size case	Total sample size control	Sample size male case	Sample size female case	Sample size male control	Sample size femalecontrol	Age case	Age control	Follow up months	ROB	Key comments
Fladt J et al., (24)	Switzerland	RCT	Multicenter	MRI/MRA	CT/CTTA	Thrombolysis, thrombectomy	200	205	95	105	101	104	72 (63–81)	73 (64–81)	3	Low	MRI group had greater 90-day independence (65.5% vs. 56.4%, aOR 1.65, *p* = 0.024) with similar sICH (2.5% vs. 3.5%) and mortality (8.5% vs. 10.8%). MRI had longer DTN (~+21 min), DTP(~+17 min), DTR (~+16 min) with similar reperfusion grade.
Kamogawa N et al., (25)	Japan	Cohort (prospective)	Multicenter	MRI (DWI)	NCCT	Thrombolysis, thrombectomy	217	126	130	87	83	43	74 (66–82)	75 (67–83)	3	Low	MRI cohort had fewer on anticoagulation and arrived later after onset, with lower NIHSS and ASPECTS. EVT was used less often in the MRI group. LKW to hospital arrival time was increased in the MRI DWI group (*p* = 0.01).
																	No significant difference was noted in Hospital arrival to IVT initiation between both groups as well as for hospital arrival to arterial puncture time. At 90-day mRS, sICH, mortality, and recanalization rates were comparable with no significant difference.
Krebs S et al., (7)	Austria	Cohort (prospective)	Multicenter	MRI	CT	Thrombolysis, thrombectomy	2,599	16,449	8,599	7,850	1,367	1,232	IVT only 75 (65–83) MT only 73 (62–80)	IVT only 72 (62–81) MT Only 71 (58–78)	3	High	CT was used more often (87% vs. 13% MRI). CT had higher sICH (4% vs. 2.8%) but shortened IVT DTN times (45 vs. 62 min *P* < 0.001). MRI added roughly 20 min to thrombectomy initiation (105 vs. 124 min, *P* < 0.001), and also
																	added 30 min for IVT initiation (120 vs. 150 min, *P* < 0.001). MRI showed higher 3-mo mRS 0–2 and lower mortality, though differences lost significance after adjustment. Difference in sICH rate was not statistically significant.
Li J et al., (26)	China	Cohort (retrospective)	Single Center	MRI	CT	Thrombolysis	172	290	93	79	151	139	70 (60–75)	69 (60–76)	3	Moderate	MRI was associated with a lower 7 and 30-day mortality, as well as lower sICH after controlling for potential confounding factors; however no difference was observed in 3-months mRS 0–2 and OTT. DNT was higher in the MRI group (51 vs. 59, *P* < 0.01).
Stösser S et al., (27)	Germany	Cohort (prospective)	Multicenter	MRI	CT	Thrombolysis, thrombectomy	370	4,268	188	182	2,101	2,167	74 (63–81)	76 (66–82)	3	Moderate	Patients in the MRI group were younger, had lower NIHSS scores, and experienced longer onset-to-admission times. Other time intervals not dependent on admission-to-imaging were not significantly different. MRI use delayed imaging by approximately 9 min but did not impact subsequent EVT workflow or 90-day outcomes. Rates of successful recanalization were similar between groups.
Atchaneeyasakul K et al., (28)	United States	Cohort	Single center	MRI	CT	Thrombolysis	66	40	28	38	20	20	72.5 ± 15.55	71.31 ± 16.68	NA	Low	MRI provided greater anatomical/physiological detail without loss of effectiveness, though CT was slightly faster. LKW to door times were similar in 2012–2014 but longer for MRI in 2015. Door to imaging times were comparable; only door to puncture and LKW to puncture time were shorter for CT. No significant outcome differences, but MRI showed a trend toward higher mRS 0–2 and lower in-hospital mortality; sICH, mortality, and reperfusion rates were similar.
Provost C et al., (29)	France	RCT	Multicenter	MRI	CT	Thrombectomy	299	102	156	143	59	43	67 (54–74)	65 (54–74)	3	Moderate	MRI provided comparable 90-day outcomes and workflow efficiency to CT. Although MRI scans were longer than CT, the interval from imaging start to IVT initiation was shorter with MRI. No significant differences were observed in rates of symptomatic intracerebral hemorrhage and 3-month mortality or successful reperfusion.
Hansen C et al., (30)	Denmark	QRCT	Single center	MRI	CT	Thrombolysis, thrombectomy	33	107	NA	NA	NA	NA	NA	NA	3	Moderate	MRI increased physician certainty in iv-tPA decisions compared to CT, without affecting 3-month mortality or functional outcomes;
																	symptomatic ICH was low and similar, indicating both imaging modalities are safe for guiding acute stroke thrombolysis. DNT was lower for CT group. The use of IVT was more certain in MRI group than CT group but lost when adjusted. No significant results for mortality, mRS and sICH.
Kang DW et al., (31)	United States	Cohort	Single center	MRI	CT	Thrombolysis	39	23	NA	NA	NA	NA	NA	NA	3	Moderate-high	MRI screening had longer DTIT and comparable ITNT, DTNT was longer for MRI. For mRS 0–1 and mRS 0–2 was significantly higher in MRI group than CT group, no difference in sICH,
																	no comparison in mortality and successful reperfusion were reported.
Solling C et al., (32)	Denmark	Cohort	Single center	MRI	CT	Thrombolysis	40	17	Overlap population with 22, 22 is more recent	Overlap population with 22, 22 is more recent	Overlap population with 22, 22 is more recent	Overlap population with 22, 22 is more recent	Overlap population with 22, 22 is more recent	Overlap population with 22, 22 is more recent	3	High	There was not significant difference in time measurements, except for imaging, MRI resulted in longer imaging time, but did not cause significant delays in DNT, imaging to needle was shorter for MRI
Solling C et al., (33)	Denmark	Cohort	Single center	MRI	CT	Thrombolysis	83	29	55	28	23	6	68 (60–73)	64 (59–73)	3	Moderate	DNT time was longer for MRI, but ONT was longer in CT group, imaging time was longer in MRI. No difference in mortality, mRS and successful reperfusion were reported.
Schellinger P et al., (34)	USA	Cohort	Multicenter	MRI	CTA	Thrombolysis	496	714	NA	NA	NA	NA	< 3 h 67 (19–91) >3 h 68.5 (26–91)	69 (21–94)	3	Moderate	MRI-based thrombolysis showed no difference in safety and effectiveness when compared to CT-based thrombolysis within 3 h, and demonstrated superior safety and efficacy beyond 3 h, without causing significant treatment delays
Köhrmann M et al., (35)	Germany	Cohort	Single center	MRI	CTA	Thrombolysis	173	209	108	65	119	90	< 3 h 70 (62–76) >3 h 70 (62–76)	73 (65–79)	3	Moderate	sICH was lower in MRI-treated patients. Functional independence did not differ significantly between MRI or CT-based reperfusion groups. Mortality was lower in the MRI group, though this difference was not statistically significant. DTN times and successful reperfusion rates were not reported.

ASPECTS, Alberta Stroke Program Early CT Score; aOR, adjusted odds ratio; CT, Computed Tomography; CTA, Computed Tomography Angiography; CTTA, Computed Tomography with Targeted Angiography; DNT, Door-to-Needle Time; DTIT, Door-to-Imaging Time; DTN, Door-to-Needle; DTP, Door-to-Puncture; DTR, Door-to-Reperfusion; DWI; Diffusion-Weighted Imaging; EVT; Endovascular Therapy; ICH, Intracerebral Hemorrhage; ITNT, Imaging-to-Needle Time; IVT, Intravenous Thrombolysis; iv-tPA, intravenous tissue Plasminogen Activator; LKW, Last Known Well; MRI, Magnetic Resonance Imaging; MRA, Magnetic Resonance Angiography; mRS, modified Rankin Scale; MT, Mechanical Thrombectomy; NA, Not Available; NCCT, Non-Contrast Computed Tomography; NIHSS, National Institutes of Health Stroke Scale; ONT, Onset-to-Needle Time; OTT, Onset-to-Treatment Time; QRCT, Quasi-Randomized Controlled Trial; RCT, Randomized Controlled Trial; ROB, Risk of Bias; sICH, symptomatic Intracerebral Hemorrhage.

### 3.3 Risk of bias

**Figure 2:**
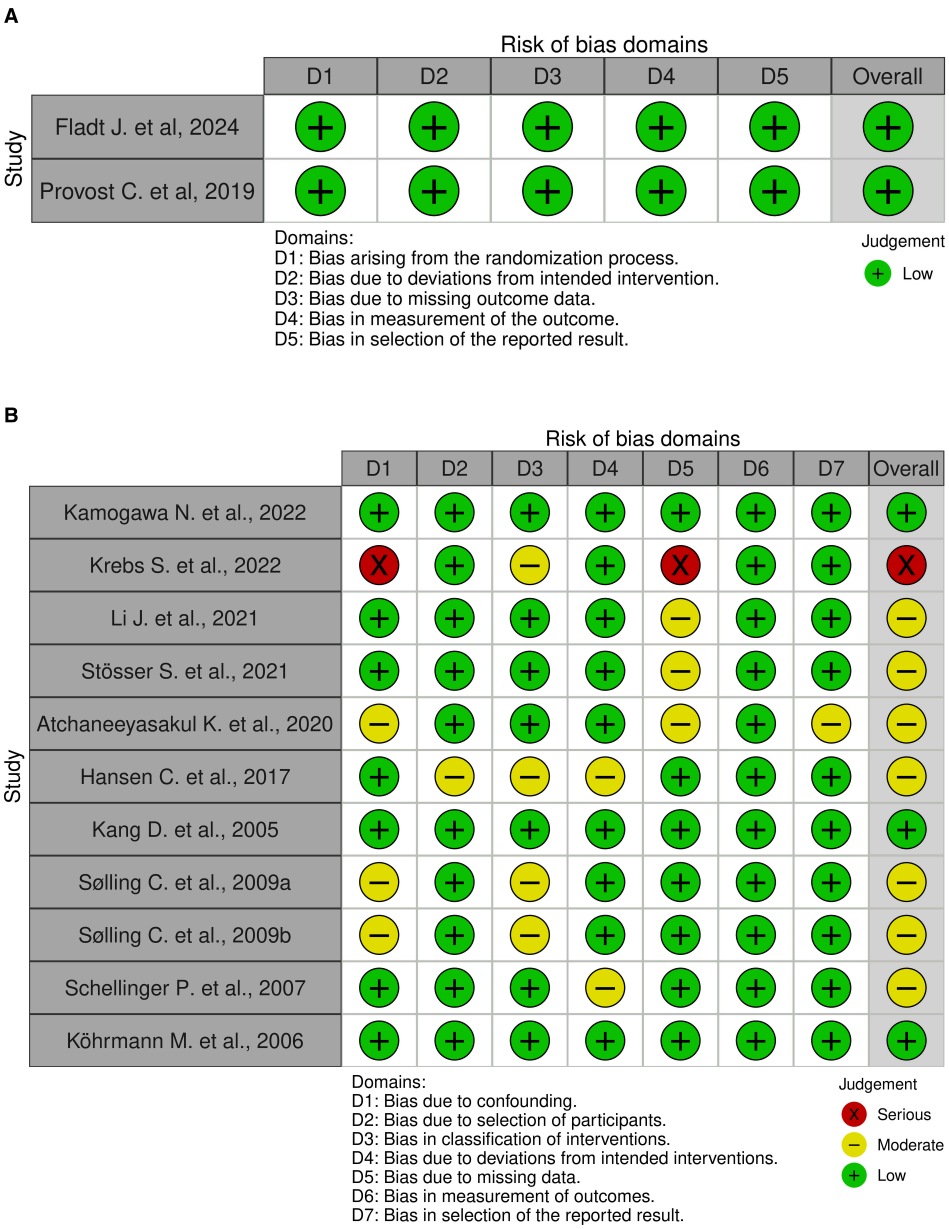
Risk of bias assessment for included randomized and non-randomized controlled trials (RoB 2.0 and ROBINS-I Tool) **(A)** Each domain represents a component of bias assessed according to the Cochrane RoB 2.0 tool. Green indicates low risk, yellow indicates some concerns, and red indicates high risk of bias **(B)** Each domain represents a component of bias assessed according to the bias ROBINS-I (Risk of Bias in non-Randomized Studies—of Interventions) tool. Green indicates low risk of bias, yellow indicates moderate risk, and red indicates serious risk of bias.

Of the 13 included studies, 11 non-randomized studies assessed with ROBINS-I showed low risk in 3 (27%), some concerns in 7 (63%), and serious risk in 1 (9%) (Figure 2B). The 2 RCTs evaluated with RoB 2.0 both demonstrated low risk of bias (Figure 2A).

### 3.4 Meta analysis

#### 3.4.1 Successful reperfusion:

Five studies with 5,893 participants (MRI *n* = 1,152; CT *n* = 4,741) were included. Random-effects model (REM) showed a pooled RR of 1.00 (95% CI: 0.91 to 1.10; *p* = 0.98; I^2^ = 55.5%; Supplementary Figure 1A). Prediction interval ranged from 0.85 to 1.18. Funnel plot shows asymmetry suggesting publication bias (Supplementary Figure 2A). Eggers Test was not performed due to low number of studies. Subgroup analysis based on risk of bias, reperfusion therapy and modality showed no significant differences between groups (*p* = 0.78, *p* = 0.13 and *p* = 0.12 respectively; Supplementary Table 11). Stratifying by study design showed statistically significant differences between subgroups (*p* < 0.0001; Supplementary Table 11). RCTs reported a slightly higher rate of successful reperfusion in the MRI group (RR = 1.08, 95% CI: 0.91 to 1.28, I^2^ = 0%), whereas cohort studies demonstrated a small, non-significant reduction (RR = 0.97, 95% CI: 0.88 to 1.07, I^2^ = 0%). Influence analysis identified Fladt J. et al., (24) as an influential, its exclusion reduced heterogeneity and slightly shifted the pooled estimate (RR = 0.97, 95% CI: 0.91 to 1.03, I2 = 0%, *p* = 0.19; Supplementary Table 14).

#### 3.4.2 90-day mRS

Meta-analysis included two studies with 246 participants (MRI *n* = 99; CT *n* = 147). REM yielded a MD of −0.61 (95% CI: −1.40 to 0.18; *p* = 0.13; I^2^ = 58.6%; Supplementary Figure 1B). The prediction interval ranged from −8.19 to 6.97. Funnel plot showed symmetry, in the presence of heterogeneity probably due to low number of studies (Supplementary Figure 2B). Egger's Test was not performed due to low number of studies. When stratified by risk of bias, subgroup differences were not statistically significant (*p* = 0.12; Supplementary Table 12). Similarly, when analyzed by study type, no statistically significant subgroup difference was found (*p* = 0.12). For reperfusion therapy and CT-modality, subgroup analyses were not perfomed because all studies were in one subgroup. Influence analysis identified Atchaneeyasakul K. et al., (28) and Hansen C. et al., (30) as influential, with their exclusion reducing heterogeneity and narrowing confidence intervals (MD = −0.55, 95% CI: −1.09 to −0.01; I^2^ = 35.2%; Supplementary Table 15).

#### 3.4.3 MRS ≤ 1

Meta-analysis five studies with 27,197 patients (MRI *n* = 4,664; CT *n* = 22,533. REM showed a pooled RR of 1.09 (95% CI: 0.94 to 1.27; *p* = 0.19; I^2^ = 0.0%; Supplementary Figure 1C), with a prediction interval of 0.92 to 1.29. Funnel plot showed symmetry indicating no publication bias (Supplementary Figure 2C). Egger's Test was not performed because of low quantity of studies. Sensitivity analysis and influence analysis were not performed due to low heterogeneity and number of studies.

#### 3.4.4 MRS ≤ 2

Meta-analysis 11 studies with 27,197 patients (MRI *n* = 4,664; CT *n* = 22,533. The pooled RR was 1.28 (95% CI: 1.06 to 1.55; *p* = 0.01; I^2^ = 82.6%; Supplementary Figure 1D), with a prediction interval from 0.72 to 2.29. Funnel plot showed asymmetry, suggesting publication bias (Supplementary Figure 2D). Egger's Test was performed with statistically significance (< 0.0001) for publication bias. Subgroup analyses were conducted according to risk of bias, study design, reperfusion therapy type and CT-modality. Across all subgroups, not statistically significant between-group differences were detected for either variable (*p* = 0.32; *p* = 0.57; *p*= 0.52; *p* = 0.16 respectively; Supplementary Table 13). Influence analysis identified Li J, et al., (26) as influential, significantly impacting pooled RR. After its exclusion, heterogeneity decreased with a consistent pooled estimate (RR = 1.24, 95% CI: 1.05 to 1.48; I^2^ = 0%; Supplementary Table 16).

#### 3.4.5 Symptomatic intracranial hemorrhage

Meta-analysis included ten studies with 27,135 patients (MRI *n* = 4,625; CT *n* = 22,510. REM yielded a pooled RR of 0.59 (95% CI: 0.45 to 0.78; *p* = 0.002; I^2^ = 7.6%; Supplementary Figure 1E), with a prediction interval from 0.38 to 0.93. Funnel plot showed symmetry (Supplementary Figure 2E). Egger's Test results confirm no asymmetry in the study (*p* = 0.71). Sensitivity and influence analysis were not performed because of low heterogeneity.

#### 3.4.6 Any intracranial hemorrhage

Meta-analysis included four studies with 5,526 patients (MRI *n* = 820; CT *n* = 4,706). The pooled estimated of REM is RR of 0.92 (95% CI: 0.77 to 1.10; *p* = 0.24; I^2^ = 0.0%; Supplementary Figure 1F), with a prediction interval from 0.68 to 1.24. Funnel plot showed symmetry, suggesting no publication bias (Supplementary Figure 2F). Due to low heterogeneity and number of studies, sensitivity and influence analysis were not performed.

#### 3.4.7 Mortality

Meta-analysis included seven studies with 7,519 patients (MRI *n* = 1,788; CT *n* = 5,731). REM showed a pooled RR of 0.72 (95% CI: 0.62 to 0.84; *p* = 0.002; I^2^ = 0.0%; Supplementary Figure 1G), with a prediction interval of 0.59 to 0.87. Funnel plot shows symmetry, indicating no publication bias (Supplementary Figure 2G). Sensitivity and influence analysis were not performed due to low heterogeneity between studies.

#### 3.4.8 Hospital mortality

Meta-analysis included two studies with 4,744 patients (MRI *n* = 436; CI *n* = 4,308). REM yielded a pooled RR of 0.71 (95% CI: 0.55 to 0.93; *p* = 0.039; I^2^ = 0.0%; Supplementary Figure 1H), with a prediction interval from 0.12 to 4.20. Funnel plot showed symmetry, suggesting no publication bias (Supplementary Figure 2H). Egger's Test was not performed because of low number of studies. Sensitivity and influence analysis were not performed because of low heterogeneity and number of studies.

## 4 Discussion

In this analysis of more than 27,000 patients, MRI-R strategies were associated with improved clinical outcomes compared with CT-based selection in AIS. MRI use was associated with significantly reduced all-cause mortality and risk of sICH while maintaining comparable rates of successful reperfusion and overall intracranial hemorrhage. Functional recovery also favored MRI guidance, with a 28% higher likelihood of achieving functional independence, although heterogeneity across studies was substantial. Given the predominance of observational studies and differences in baseline characteristics across included studies, these findings should be interpreted as hypothesis-generating rather than definitive MRI superiority. These results may reflect MRI's superior tissue characterization allows more accurate identification of viable brain tissue, which could partly explain the lower hemorrhagic complications and better functional outcomes in the MRI-selected cohort.

CT and MRI differ fundamentally in how they guide reperfusion therapy. CT-based protocols, typically beginning with NCCT, are designed for rapid exclusion of intracranial hemorrhage and gross infarction, enabling prompt initiation of IVT within minutes of arrival. When supplemented with CTA and CTP, these protocols can identify vessel occlusion and delineate core–penumbra mismatch, supporting MT decisions (36). In contrast, MRI-guided approaches rely on DWI to define the infarct core and PWI to evaluate tissue at risk (37). This combination allows precise differentiation between irreversibly damaged and salvageable tissue, even beyond conventional therapeutic windows. Additional MRI sequences like GRE or SWI, further aid in detecting microbleeds and hemorrhagic risk before thrombolysis (38). MRI provides a more comprehensive physiological assessment, while CT prioritizes speed and accessibility (7). The choice between modalities therefore reflects a balance between diagnostic precision and time sensitivity.

With this in mind, comparing MRI- and CT-based selection is clinically important because the two modalities differ markedly in their ability to identify infarct core, estimate hemorrhagic risk, and guide safe reperfusion. Historically, CT has been the gold standard at the time of reperfusion because it is the fastest and most widely available imaging approach. However, although we observed that MRI-based triage extended door-to-imaging time, it was still associated with a significant mortality benefit (RR 0.72), consistent with the findings of a previous meta-analysis by Bankole et al., who reported a 41% reduction in mortality among EVT patients selected with MRI (39). This advantage may be explained by MRI's superior ability to define infarct core, detect blood–brain barrier injury, and assess tissue viability. These characteristics also help explain the robust 41% reduction in symptomatic ICH (RR 0.59, 95% CI: 0.45 to 0.78; *p* = 0.002; I^2^ = 7.6%) compared with CT-based selection in our low-heterogeneity population. Notably, this symptomatic ICH reduction contrasts with the previous meta-analysis by Bankole et al., who did not observe significant differences before and after adjustment (OR 0.39, 95 % CI: 0.13–1.15, 3 studies, I^2^ =75 %, *p* = 0.02) (39), This difference in results may be attributed to a larger sample size and narrower CI in our study, suggesting a more precise effect. Our safety findings align with the mechanistic observations from a meta-analysis by Adebayo et al., which included 12 studies and 808 AIS patients, who demonstrated that advanced imaging modalities capable of characterizing core, hypoperfusion, and blood–brain barrier permeability exhibit high sensitivity (≈86%) for predicting hemorrhagic transformation (40). However, these explanations are based on indirect evidence, and analyses using predominantly retrospective and observational data are insufficient to establish causality. Another important finding is the improvement in functional capacity: patients selected with MRI were 28% more likely to achieve functional independence, an outcome association not described in previous reviews. Regarding reperfusion success, no significant differences were identified between MRI- and CT-R, suggesting that imaging modality influences patient selection rather than procedural efficacy. Our findings align with current American Heart Association/ American Stroke Association and European Stroke Organization guidelines, which endorse CT as the first-line modality for rapid hemorrhage exclusion but permit MRI when it does not delay treatment (41, 42). By demonstrating lower mortality, reduced symptomatic ICH, and greater functional independence with MRI-based selection, our results strengthen current recommendations. The 2018 American Heart Association/American Stroke Association guidelines classified routine MRI use for acute stroke selection as Class IIb, B-NR, and for secondary stroke prevention as IIa, C-EO, largely based on limited data and expert consensus. Our meta-analysis, combining RCTs and observational studies, provides updated quantitative support for a clinically meaningful association between MRI-R and improved functional and safety outcomes (42). However, these findings should be interpreted with caution. Baseline stroke severity showed a non-significant trend favoring MRI across several included studies, reaching statistical significance in some studies like Kamogawa et al. (median 16 vs. 20, *P* < 0.01), who also reported significantly less frequent premorbid anticoagulation in the MRI group (17.5% vs. 27.8%, *P* = 0.02) (7, 24, 25, 28, 30, 32, 33). Across studies, CT was consistently used as the fallback modality for patients with MRI contraindications, late presentations, unstable medical conditions, obesity, claustrophobia, or pacemakers, with clinically unstable patients systematically shifted to CT. Stösser et al. further demonstrated that MRI was used in only 8% of EVT patients, with 73.5% concentrated in just three of 25 centers, suggesting MRI use was driven by center-level expertise and selective patient triaging rather than routine practice (27). Collectively, these factors are likely to have introduced selection bias and confounding by indication favoring MRI, and may have independently contributed to the better outcomes observed. Supporting this, Provost et al., which enrolled a more balanced population through strict NIHSS-based inclusion criteria, did not find MRI to be independently associated with superior functional outcomes in multivariable analysis (29). These are recognized limitations of observational studies, and highlight why the observed associations should not be interpreted as definitive superiority.

The 90-day mRS ≤ 2 showed substantial heterogeneity (I^2^ = 82.6%, PI 0.72–2.29). The wide prediction interval indicates that while the pooled estimate favors MRI-R, the true effect in any individual population may range from a modest harm to a substantial benefit, reflecting genuine variability in patient selection, imaging protocols, and center-level expertise across included studies. Differences in baseline NIHSS, treatment time window, reperfusion strategy, and thrombolysis dosing are likely to have contributed to this heterogeneity. Influence analysis identified Li J et al. as the primary driver (26). Despite comparable baseline NIHSS scores, the MRI group exhibited worse baseline imaging profiles, with lower median ASPECTS (8 vs. 9, *P* < 0.01) and a greater proportion of patients with ASPECTS < 7 (25.6% vs. 9.7%). This likely reflects MRI's superior lesion detection sensitivity rather than genuinely greater stroke severity. Lower ASPECTS inherently indicates reduced salvageable brain tissue, which may have limited functional independence in the MRI group thereby attenuating the difference between both imaging groups and contributing to the absence of a significant difference in 90-day mRS ≤ 2 (OR 1.32, 95% CI 0.93–1.84, *P* = 0.71). Upon its omission, I^2^ reduced to 0% with a pooled effect of 1.15 (95% CI 1.09–1.22), suggesting the association between MRI-guided selection and improved functional outcomes is more consistent across comparable populations than the primary analysis implies. Subgroup analyses across risk of bias, study design, and reperfusion therapy type showed no statistically significant between-group differences, further suggesting the direction of association remained consistent irrespective of these sources of heterogeneity. Egger's test was statistically significant (*p* < 0.0001) and the funnel plot demonstrated asymmetry, suggesting possible publication bias or small-study effects. Among the smallest included studies, Kang DW et al., Provost C et al., and Li J et al. reported some of the largest risk ratios (1.70, 1.36, and 2.73, respectively) (26, 29, 31). Kang DW et al. operated a dedicated 24-h MRI center, Provost C et al. drew from a trial where 74% were MRI-selected, and Li J et al. enrolled patients with paradoxically worse baseline ASPECTS in the MRI group, all likely amplifying their effect estimates. These findings may suggest that smaller studies conducted in highly selected or MRI-optimized healthcare centers may have contributed disproportionately to the pooled effect estimates.

NCCT, while fast and widely available, has a sensitivity of only 60% for acute ischemia even in moderate-to-severe stroke within the first 6 h of onset, limiting its ability to characterize infarct core and penumbra. CTA and CTP substantially improve diagnostic sensitivity and provide additional information on ischemia severity, location, and extent, but are implemented variably across stroke centers (43, 44). Comparing MRI-R against plain NCCT is not directly comparable to comparing it against modern CTP, which itself can delineate penumbra-core mismatch with reasonable accuracy (45). The apparent MRI advantages observed in older studies within our analysis may therefore partially reflect the limitations of the CT comparator used rather than a true superiority of MRI-R. Subgroup analyses comparing NCCT-only vs. MRI and multimodal CT vs. MRI did not reveal statistically significant differences for successful reperfusion and 90-day mRS ≤ 2, with the direction of effect consistently favoring MRI across both CT comparator groups. This suggests the observed association is not dependent on a single CT imaging modality. Similarly, subgroup analysis by reperfusion type (IVT, EVT or both) showed no statistically significant differences in successful reperfusion and 90-day mRS ≤ 2, suggesting that the benefit of MRI guidance is consistent across treatment strategies and likely reflects improved patient selection rather than an advantage specific to any one reperfusion approach. Together, these findings collectively support the robustness of the primary analysis, though they should be interpreted cautiously, as few studies per group limit statistical power to detect real differences, highlighting the need for larger, prospectively defined comparisons to clarify whether the degree of benefit varies by CT imaging modality or reperfusion type.

Though MRI-R theoretically demonstrates some functional and safety advantages, its feasibility in non-tertiary or resource-limited settings may pose a challenge. Increased workflow times associated with MRI-R may outweigh the theoretical benefits in time-sensitive management of stroke patients. In centers without optimized MRI protocols, dedicated stroke teams, or 24/7 scanner and operator availability, these delays may limit MRI-R as a routine strategy.

From a clinical standpoint, imaging selection should therefore be individualized rather than uniformly applied. CT-R remains the cornerstone of hyperacute stroke management given its speed, availability, and reliability in excluding hemorrhage. MRI-R was associated with better outcomes, potentially reflecting the value of more detailed tissue characterization when it can be achieved without delaying reperfusion. However, whether this reflects a true imaging-based benefit or residual selection of more favorable patients cannot be established definitively, an uncertainty explained by the observational design of most included studies, baseline patient characteristic differences, and publication bias as detailed above. Hence these findings should not be interpreted as definitive superiority of MRI-based reperfusion over CT, but as hypothesis-generating evidence highlighting the need for further prospective, RCTs in appropriately equipped centers.

### 4.1 Strengths

Our study possesses several notable strengths. It integrates data from over 27,000 patients, encompassing both IVT and MT cohorts, thereby enhancing generalizability across reperfusion strategies. The inclusion of diverse geographic regions and study designs, along with comprehensive outcome assessment, including mortality, functional independence, and hemorrhagic complications, provides a balanced and clinically meaningful overview of imaging-based selection in AIS. Where feasible, subgroup analyses stratified by study type, reperfusion strategy, and CT-based imaging modality allowed exploring outcome consistency across treatment strategies.

### 4.2 Limitations

Several limitations should be recognized. Most included studies were observational and retrospective, limiting causal inference and introducing potential selection bias. Variability in CT comparator protocols (NCCT to multimodal CTP) and the absence of standardized imaging criteria limited direct comparability. Studies spanned nearly two decades of evolving AIS management, including the adoption of EVT and changing thrombolysis protocols, introducing temporal confounding that aggregate data could not fully adjust for. Inconsistent time metrics and limited patient-level data constrained subgroup analyses. Pooled estimates were derived from aggregate outcomes; confounders varied widely across studies, precluding pooling of adjusted estimates and leaving residual confounding by indication possible. Publication bias could not be excluded for outcomes with few studies and, where detected, may have overestimated the benefit of MRI-R. The number of RCTs remains limited.

Baseline severity differences further limit causal inference. MRI-selected patients may have presented later, been more stable, or been triaged to better-resourced centers, representing confounding by indication that observational designs cannot adequately control. These associations should not be interpreted as causal.

The inability to stratify outcomes by occlusion location is an additional limitation, as proximal and distal occlusions differ in severity, reperfusion success, and hemorrhagic risk. Occlusion site, along with baseline NIHSS, onset-to-treatment times, and imaging-to-reperfusion intervals, were inconsistently reported across studies, precluding adjustment for these variables.

Lastly, although leave-one-out sensitivity analysis was performed, the DerSimonian-Laird random-effects method was used without Hartung-Knapp-Sidik-Jonkman adjustment, which may underestimate uncertainty when heterogeneity is substantial or few studies contribute to an analysis (46). Therefore, confidence intervals from subgroup analyses with few contributing studies should be interpreted with caution.

### 4.3 Future recommendations

Future research should focus on high-quality, prospective, multicenter trials directly comparing MRI- and CT-R pathways under standardized workflow conditions. Integration of advanced imaging biomarkers, such as perfusion mismatch ratios, collateral grading, and blood–brain barrier permeability, may further clarify which patients benefit most from MRI-guided selection. Additionally, cost-effectiveness and feasibility analyses across diverse healthcare systems will be essential to determine the real-world applicability of MRI-based approaches.

## 5 Conclusion

This SR and meta-analysis suggests that the use of MRI-R was associated with improved functional outcomes, reduced mortality, and lower rates of sICH compared to CT-guided approaches. However, these findings should be interpreted with caution given the inclusion of predominantly observational studies and baseline differences between imaging groups. Imaging selection should remain context dependent. In the hyperacute setting, CT-R continues to represent the standard of care due to its speed, broad availability and reliability in excluding intracranial hemorrhage, allowing rapid initiation of therapy. When available, MRI-R provides detailed tissue characterization which may aid patient selection, though its benefit over CT-based approaches remains unconfirmed. Continued investigation through prospective, multicenter studies will be essential to validate these associations and to delineate for the optimal role of MRI-R within stroke care pathways.

## Data Availability

The original contributions presented in the study are included in the article/Supplementary material, further inquiries can be directed to the corresponding author.
