# Supplementary material for: Efficacy and safety of magnetic resonance imaging- vs. computed tomography-guided reperfusion in acute ischemic stroke: a systematic review and meta-analysis
Source: Front Neurol. 2026 Aug 3;17:1862054. doi: 10.3389/fneur.2026.1862054 (PMC13478007; doi:10.3389/fneur.2026.1862054)
Supplement: Supplementary file 1 [file Table_1.docx]

Supplementary Material

# 1 Supplementary Figures and Tables

## 1.1 Supplementary Figures

**Supplementary Figure 1:** Forest plots of risk ratios for outcomes and adverse events comparing MRI vs CT-based reperfusion. (A) Successful reperfusion (B) 90-day mRS (C): mRS ≤1 (D): mRS ≤2 (E) Symptomatic intracranial hemorrhage (F): Any intracranial hemorrhage (G): Mortality (H): Hospital Mortality

**Supplementary Figure 2:** Funnel plots assessing publication bias for various outcomes comparing MRI vs CT-based reperfusion in adults with acute ischemic stroke (A) Successful reperfusion (B) 90-day mRS (C) mRS ≤1 (D): mRS ≤2 (E) Symptomatic Intracranial Hemorrhage (F): Any Intracranial Hemorrhage (G) Mortality (H) Hospital Mortality

## 1.2 Supplementary Tables

**Supplementary Table 1. PubMed Search**

| **Search** | **Results** |
| --- | --- |
| ( (Stroke[MeSH]) OR (Brain Ischemia[MeSH]) OR (Cerebral Infarction[MeSH]) OR (Ischemic Attack, Brain[MeSH]) OR (acute ischemic stroke[Title/Abstract]) OR (acute stroke[Title/Abstract]) OR (ischemic stroke[Title/Abstract]) OR (brain ischemia[Title/Abstract]) OR (cerebral infarction[Title/Abstract]) OR (cerebral ischemia[Title/Abstract]) ) AND ( (Magnetic Resonance Imaging[MeSH]) OR (Diffusion Magnetic Resonance Imaging[MeSH]) OR (MRI[Title/Abstract]) OR (magnetic resonance imaging[Title/Abstract]) OR (diffusion weighted imaging[Title/Abstract]) OR (DWI[Title/Abstract]) OR (perfusion MRI[Title/Abstract]) OR (mismatch imaging[Title/Abstract]) OR (MRI-based selection[Title/Abstract]) OR (MRI-guided therapy[Title/Abstract]) ) AND ( (Tomography, X-Ray Computed[MeSH]) OR (CT[Title/Abstract]) OR (computed tomography[Title/Abstract]) OR (noncontrast CT[Title/Abstract]) OR (CT-based selection[Title/Abstract]) OR (CT-guided therapy[Title/Abstract]) OR (CT perfusion[Title/Abstract]) OR (NCCT[Title/Abstract]) ) AND ( (Thrombolytic Therapy[MeSH]) OR (Thrombectomy[MeSH]) OR (reperfusion therapy[Title/Abstract]) OR (mechanical thrombectomy[Title/Abstract]) OR (endovascular thrombectomy[Title/Abstract]) OR (thrombolysis[Title/Abstract]) OR (tPA[Title/Abstract]) OR (alteplase[Title/Abstract]) OR (tenecteplase[Title/Abstract]) OR (clot retrieval[Title/Abstract]) OR (stroke intervention[Title/Abstract]) ) | 1682 |

**Supplementary Table 2: EMBASE Search**

| **Search** | **Results** |
| --- | --- |
| (  'stroke'/exp OR  'brain ischemia'/exp OR  'cerebral infarction'/exp OR  'brain ischemic attack'/exp OR  'acute ischemic stroke':ti,ab OR  'acute stroke':ti,ab OR  'ischemic stroke':ti,ab OR  'brain ischemia':ti,ab OR  'cerebral infarction':ti,ab OR  'cerebral ischemia':ti,ab  )  AND  (  'magnetic resonance imaging'/exp OR  'diffusion magnetic resonance imaging'/exp OR  mri:ti,ab OR  'magnetic resonance imaging':ti,ab OR  'diffusion weighted imaging':ti,ab OR  dwi:ti,ab OR  'perfusion mri':ti,ab OR  'mismatch imaging':ti,ab OR  'mri-based selection':ti,ab OR  'mri-guided therapy':ti,ab  )  AND  (  'computed tomography'/exp OR  ct:ti,ab OR  'computed tomography':ti,ab OR  'noncontrast ct':ti,ab OR  'ct-based selection':ti,ab OR  'ct-guided therapy':ti,ab OR  'ct perfusion':ti,ab OR  ncct:ti,ab  )  AND  (  'thrombolysis'/exp OR  'thrombectomy'/exp OR  'reperfusion therapy':ti,ab OR  'mechanical thrombectomy':ti,ab OR  'endovascular thrombectomy':ti,ab OR  thrombolysis:ti,ab OR  tpa:ti,ab OR  alteplase:ti,ab OR  tenecteplase:ti,ab OR  'clot retrieval':ti,ab OR  'stroke intervention':ti,ab  ) | 7317 |

**Supplementary Table 3. Web of Science Search**

| **Search** | **Results** |
| --- | --- |
| TS=(  "acute ischemic stroke" OR  "acute stroke" OR  "ischemic stroke" OR  "brain ischemia" OR  "cerebral infarction" OR  "cerebral ischemia" OR  "stroke"  )  AND TS=(  "MRI" OR  "magnetic resonance imaging" OR  "diffusion weighted imaging" OR  "DWI" OR  "perfusion MRI" OR  "mismatch imaging" OR  "MRI-based selection" OR  "MRI-guided therapy"  )  AND TS=(  "CT" OR  "computed tomography" OR  "noncontrast CT" OR  "CT-based selection" OR  "CT-guided therapy" OR  "CT perfusion" OR  "NCCT"  )  AND TS=(  "reperfusion therapy" OR  "mechanical thrombectomy" OR  "endovascular thrombectomy" OR  "thrombolysis" OR  "tPA" OR  "alteplase" OR  "tenecteplase" OR  "clot retrieval" OR  "stroke intervention"  ) | 1653 |

**Supplementary Table 4. CINAHL Search**

| **Search** | **Results** |
| --- | --- |
| (  "acute ischemic stroke" OR  "acute stroke" OR  "ischemic stroke" OR  "brain ischemia" OR  "cerebral infarction" OR  "cerebral ischemia" OR  "stroke"  )  AND (  "MRI" OR  "magnetic resonance imaging" OR  "diffusion weighted imaging" OR  "DWI" OR  "perfusion MRI" OR  "mismatch imaging" OR  "MRI-based selection" OR  "MRI-guided therapy"  )  AND (  "CT" OR  "computed tomography" OR  "noncontrast CT" OR  "CT-based selection" OR  "CT-guided therapy" OR  "CT perfusion" OR  "NCCT"  )  AND (  "reperfusion therapy" OR  "mechanical thrombectomy" OR  "endovascular thrombectomy" OR  "thrombolysis" OR  "tPA" OR  "alteplase" OR  "tenecteplase" OR  "clot retrieval" OR  "stroke intervention"  ) | 390 |

**Supplementary Table 5. Google Scholar Search**

| **Search** | **Results** |
| --- | --- |
| ("acute ischemic stroke" OR "ischemic stroke" ) AND ("MRI" OR "magnetic resonance" OR "DWI" OR "perfusion MRI") AND ("CT" OR "computed tomography" ) AND ("thrombolysis" OR "thrombectomy" OR "reperfusion therapy" OR "tPA" OR "alteplase" OR "tenecteplase") | 200 |

**Supplementary Table 6. Cochrane Search**

| **ID** | **Search** | **Hits** |
| --- | --- | --- |
| #1 | MeSH descriptor: [Stroke] explode all trees | 18521 |
| #2 | MeSH descriptor: [Brain Ischemia] explode all trees | 5910 |
| #3 | MeSH descriptor: [Cerebral Infarction] explode all trees | 1759 |
| #4 | Ischemic Attack, Brain | 1301 |
| #5 | ("acute ischemic stroke" OR "ischemic stroke" OR "acute stroke" OR "brain ischemia" OR "cerebral infarction" OR "cerebral ischemia"):ti,ab,kw | 24751 |
| #6 | #1 OR #2 OR #3 OR #4 OR #5 | 36606 |
| #7 | MeSH descriptor: [Magnetic Resonance Imaging] explode all trees | 13462 |
| #8 | MeSH descriptor: [Diffusion Magnetic Resonance Imaging] explode all trees | 693 |
| #9 | ("MRI" OR "magnetic resonance imaging" OR "diffusion weighted imaging" OR "DWI" OR "perfusion MRI" OR "mismatch imaging" OR "MRI-based selection" OR "MRI-guided therapy"):ti,ab,kw | 52402 |
| #10 | #7 OR #8 OR #9 | 52919 |
| #11 | MeSH descriptor: [Tomography, X-Ray Computed] explode all trees | 8987 |
| #12 | ("CT" OR "computed tomography" OR "noncontrast CT" OR "CT-based selection" OR "CT-guided therapy" OR "CT perfusion" OR "NCCT"):ti,ab,kw | 92420 |
| #13 | #11 OR #12 | 93803 |
| #14 | MeSH descriptor: [Thrombolytic Therapy] explode all trees | 2664 |
| #15 | MeSH descriptor: [Thrombectomy] explode all trees | 873 |
| #16 | ("reperfusion therapy" OR "mechanical thrombectomy" OR "endovascular thrombectomy" OR "thrombolysis" OR "tPA" OR "alteplase" OR "tenecteplase" OR "clot retrieval" OR "stroke intervention"):ti,ab,kw | 9963 |
| #17 | #14 OR #15 OR #16 | 11039 |
| #18 | #6 AND #10 AND #13 AND #17 | 336 |

**Supplementary Table 7. Reasons for Exclusion of Full-Text Articles**

| **Author** | **Paper title** | **Reason for exclusion** |
| --- | --- | --- |
| Hidayat R et al., 2025 | The Necessity of Using MRI as an Imaging Modality in Acute Code Stroke in Indonesia. | Wrong study design: all patients went through both CT and MRI without direct efficacy comparison |
| Tsuda A et al., 2025 | The comparison of MRI and CT protocol examination times for mechanical thrombectomy in acute ischemic stroke. | Wrong outcome: reports diagnostic timing only; not aligned with our outcomes |
| Ohtsubo H et al., 2024 | Magnetic Resonance Imaging versus Computed Tomography Before Mechanical Thrombectomy for Acute Ischemic Stroke | Wrong publication type: abstract |
| Ducroux C et al., 2024 | CT Or MRI protocol for acute stroke reperfusion with EVT (COMPARE): an international retrospective cohort study | Wrong study design |
| Qu H et al., 2024 | Target-based deep learning network surveillance of non-contrast computed tomography for small infarct core of acute ischemic stroke | Wrong study design |
| Rapillo CM et al., 2024 | Moving From CT to MRI Paradigm in Acute Ischemic Stroke: Feasibility, Effects on Stroke Diagnosis and Long-Term Outcomes. | Wrong study design: no imaging strategy comparison; outcomes not aligned; reperfusion subgroup not analyzed separately |
| Yoo AJ et al., 2024 | Thrombectomy for Stroke with Large Infarct on Noncontrast CT: The TESLA Randomized Clinical Trial | Wrong intervention: wrong intervention not aligned with inclusion criteria |
| Kumar M et al., 2023 | Is improved access to magnetic resonance imaging imperative for optimal ischemic stroke care? | Wrong publication type |
| Fladt J et al., 2023 | Magnetic Resonance Imaging versus Computed Tomography for Baseline Imaging Evaluation in Acute Large Artery Ischemic Stroke: a subanalysis of the SWIFT-DIRECT trial | Wrong publication type: abstract |
| Rapillo CM et al., 2023 | Moving from CT-first to MRI-first paradigm in acute ischemic stroke: effects on time metrics and revascularization rates and safety | Wrong publication type: abstract |
| Miller S et al., 2023 | Magnetic Resonance Versus Computed Tomography As Preferred Screening Of Suspected Acute Stroke: A Retrospective Comparative Cohort Analysis | Wrong publication type: abstract |
| Miao J et al., 2023 | Effect of Imaging Selection Paradigms on Endovascular Thrombectomy Outcomes in Patients With Acute Ischemic Stroke. | Wrong study design |
| Derraz I et al., 2023 | Endovascular thrombectomy outcomes with and without intravenous thrombolysis for large ischemic cores identified with CT or MRI | Wrong study design |
| Zhu C et al., 2023 | Perfusion deficits in thrombolysis-treated acute ischemic stroke patients with negative or positive diffusion-weighted imaging. | Wrong study design: compares MRI DWI-positive vs negative patients; no direct CT vs MR based reperfusion comparison, focuses on post-thrombolysis image findings |
| Cheng H et al., 2023 | Does MRI add value in selecting patients for thrombectomy beyond the 6 h window? A matched-control analysis | Wrong study design: includes patients who received reperfusion >6 hours |
| Fischer U et al., 2022 | Magnetic Resonance Imaging or Computed Tomography for Suspected Acute Stroke: Association of Admission Image Modality with Acute Recanalization Therapies, Workflow Metrics, and Outcomes. | Wrong study design and includes patients with all stroke types |
| Krishnaswamy D et al., 2022 | Predicting Imaging Outcomes in Acute Stroke Therapy-Comparison of Magnetic Resonance Imaging and Computed Tomography | Wrong study design: all patients underwent both CT and MRI prior to reperfusion |
| Nguyen TN et al., 2022 | Noncontrast Computed Tomography vs Computed Tomography Perfusion or Magnetic Resonance Imaging Selection in Late Presentation of Stroke With Large-Vessel Occlusion. | Wrong study design: includes patients who received reperfusion >6 hours |
| Porto GBF et al., 2022 | Association of Noncontrast Computed Tomography and Perfusion Modalities With Outcomes in Patients Undergoing Late-Window Stroke Thrombectomy. | Wrong study design: only includes patients who received reperfusion >6 hours |
| Slawski Md DE et al., 2021 | Cerebral Perfusion Imaging for Intravenous Thrombolysis Treatment. | Wrong publication type |
| Althaus K et al., 2021 | MRI as a first-line imaging modality in acute ischemic stroke: a sustainable concept | Wrong study design: includes TIA patients; ischemic subgroup not clearly separated |
| Meinel TR et al., 2020 | Association of initial imaging modality and futile recanalization after thrombectomy | Wrong study design |
| Sakamoto Y et al., 2020 | Reducing door-to-reperfusion time in acute stroke endovascular therapy using magnetic resonance imaging as a screening modality. | Wrong comparator: no CT-based reperfusion comparator arm |
| Fuhrer H et al., 2019 | MRI versus CT imaging for selection of mechanical thrombectomy in anterior ischemic stroke and large vessel occlusion: Effects on clinical outcome and workflow times | Wrong publication type: abstract |
| Atchaneeyasakul K et al., 2019 | Efficient multimodal-mri versus ct selection for anterior circulation large vessel | Wrong publication type: abstract |
| Liang BJ et al., 2019 | Early Analysis of Acute Cerebral Infarction by Magnetic Resonance Diffusion Weighted Imaging | Wrong comparator: no CT-based reperfusion comparator arm |
| Ringleb P et al., 2019 | Extending the time window for intravenous thrombolysis in acute ischemic stroke using magnetic resonance imaging-based patient selection | Wrong study design: only evaluates MRI-based reperfusion vs placebo (>4.5 h window) |
| Meckel S et al., 2018 | MR versus CT for selection of mechanical thrombectomy in patients with acute ischemic stroke and anterior circulation large vessel occlusion | Wrong publication type: abstract |
| Flottmann F et al., 2018 | Treatment delay of MRI vs. CT admission imaging in acute stroke triage | Wrong publication type: abstract |
| Sablot D et al., 2018 | Target door-to-needle time for tissue plasminogen activator treatment with magnetic resonance imaging screening can be reduced to 45 min | Wrong study design: MRI-only reperfusion therapy, no CT-comparator arm |
| Simonsen CZ et al., 2017 | Workflow metrics for MRI selection of patients for endovascular therapy in the goliath trial | Wrong publication type: abstract |
| Pereira D et al., 2017 | ADC quantification in basilar artery occlusion as an indicator of clinical outcome after endovascular treatment | Wrong study design |
| Hansen CK et al., 2017 | CT and MRI-based door-needle-times for acute stroke patients a quasi-randomized clinical trial. | Wrong study design and includes patients with all stroke types |
| Lansberg MG et al., 2017 | Computed tomographic perfusion to predict response to recanalization in ischemic stroke | Wrong intervention: no MRI-based reperfusion intervention arm |
| Hansen CK et al., 2016 | Physicians are more certain when prescribing thrombolysis to MRI-examined stroke patients | Wrong publication type: oral session |
| Hansen CK et al., 2016 | Door-needle-times for CT versus MRIexamined stroke patients: A clinical randomised trial | Wrong publication type: poster |
| Sakamoto Y et al., 2016 | Feasibility of using magnetic resonance imaging as a screening tool for acute stroke thrombolysis. | Wrong study design: no CT-based reperfusion arm; focused on workflow/time reduction without reporting relevant clinical outsomes |
| Hansen CK et al., 2015 | CT or MRI in evaluation for I.V. Thrombolysis: A single-center quasi randomized trial | Wrong publication type: abstract |
| Cha JK et al., 2015 | A systemized stroke code significantly reduced time intervals for using IV T-PA under MRI screening | Wrong study design |
| Tateishi Y et al., 2015 | Large deep white matter lesions may predict futile recanalization in endovascular therapy for acute ischemic stroke. | Wrong comparator: no CT-based reperfusion comparator arm |
| Shah S et al., 2015 | Screening with MRI for Accurate and Rapid Stroke Treatment SMART | Wrong comparator: only MRI-based reperfusion therapy; no CT comparison arm |
| Hansen CK et al., 2014 | On-going clinical trials computerized tomography (ct) or magnetic resonance imaging (mri) in work up for i.v. thrombolysis: A single-center quasi randomized trial | Wrong publication type |
| Wisco D et al., 2014 | Addition of hyperacute MRI AIDS in patient selection, decreasing the use of endovascular stroke therapy. | Wrong study design: all patients underwent both CT and MRI before reperfusion |
| Naik D et al., 2014 | Susceptibility weighted magnetic resonance imaging of brain: A multifaceted powerful sequence that adds to understanding of acute stroke | Wrong comparator: no CT-based reperfusion comparator arm |
| Simonsen CZ et al., 2014 | MRI before intraarterial therapy in ischemic stroke: feasibility, impact, and safety. | Wrong comparator: no CT-based reperfusion comparison |
| Kochar AS et al., 2013 | Computed Tomography or Magnetic Resonance Perfusion Imaging Benefits Selection of Acute Ischemic Stroke Patients for Endovascular Treatment | Wrong publication type: meeting report |
| Kawano H et al., 2013 | Hyperacute-phase computed tomography-diffusion-weighted imaging discrepancy and response to thrombolysis. | Wrong study design: all patients underwent both CT and MRI |
| Kawano H et al., 2013 | Diffusion-Weighted Magnetic Resonance Imaging May Underestimate Acute Ischemic Lesions Cautions on Neglecting a Computed Tomography-Diffusion-Weighted Imaging Discrepancy | Wrong study design: all patients underwent both CT and MRI |
| Hwang DY et al., 2012 | Comparative sensitivity of computed tomography vs. magnetic resonance imaging for detecting acute posterior fossa infarct. | Wrong study design: MRI-only study without CT comparator; does not assess reperfusion strategies or clinical outcomes |
| Hurley MC et al., 2012 | Neuroimaging in Acute Stroke: Choosing the Right Patient for Neurointervention | Wrong study design: non-comparative design |
| Nezu T et al., 2011 | Early ischemic change on CT versus diffusion-weighted imaging for patients with stroke receiving intravenous recombinant tissue-type plasminogen activator therapy â€¦ | Wrong study design: all patients underwent both CT and MRI; imaging comparison diagnostic, not interventional outcomes |
| Jovin TG et al., 2011 | Imaging-based endovascular therapy for acute ischemic stroke due to proximal intracranial anterior circulation occlusion treated beyond 8 hours from time last seen well: retrospective multicenter analysis of 237 consecutive patients. | Wrong study design: includes patients who received reperfusion >6 hours |
| Davis S et al., 2010 | Imaging guided patient selection: CT or MR-techniques? | Wrong publication type: abstract |
| Nagel S et al., 2010 | MR based thrombolysis in patients with acute basilar artery occlusion | Wrong publication type: abstract |
| Yoo SH et al., 2010 | Comparison between MRI screening and CT-plus-MRI screening for thrombolysis within 3 h of ischemic stroke. | Wrong study design: includes patients who underwent both CT and MRI without separate comparator groups |
| Ribo M et al., 2009 | MRI versus CT-based thrombolysis in acute stroke in the 3 to 4.5 hours time window | Wrong publication type: abstract |
| Earnshaw SR et al., 2009 | Cost-effectiveness of patient selection using penumbral-based MRI for intravenous thrombolysis. | Wrong study design |
| Kawano H et al., 2009 | Hyperacute phase CT-DWI reversed discrepancy and response to thrombolysis. | Wrong study design |
| Li YH et al., 2009 | Comparison of MRI-based thrombolysis for patients with middle cerebral artery occlusion<or=3 h and 3-6 h. | Wrong study design: lacks CT comparator; could meet inclusion criteria based on intervention/comparator definitions |
| Kim EY et al., 2006 | Reversed discrepancy between CT and diffusion-weighted MR imaging in acute ischemic stroke | Wrong study design: all patients underwent both CT and MRI |
| Barber PA et al., 2005 | Imaging of the brain in acute ischemic stroke: comparison of computed tomography and magnetic resonance diffusion-weighted imaging | Wrong study design: all patients underwent both CT and MRI |
| Fiehler J et al., 2004 | Are there time-dependent differences in diffusion and perfusion within the first 6 hours after stroke onset? | Wrong study design: no CT comparator; outcomes not aligned with inclusion criteria |
| Buckley BT et al., 2003 | Audit of a policy of magnetic resonance imaging with diffusion-weighted imaging as first-line neuroimaging for in-patients with clinically suspected acute stroke | Wrong study design: no reperfusion therapy administered |
| Jaillard A et al., 2002 | Significance of early CT signs in acute stroke. A CT scan-diffusion MRI study. | Wrong study design: CT and MRI performed on same cohort without separate comparison; no reperfusion therapy administered |
| Kitagawa N et al., 2001 | Local thrombolysis for acute ischemic stroke based on findings of diffusion and perfusion MRI. | Wrong comparator: no CT-based reperfusion comparator arm |
| Schellinger PD et al., 2000 | Feasibility and practicality of MR imaging of stroke in the management of hyperacute cerebral ischemia. | Wrong study design: no comparison between CT and MRI modalities |
| Albers GW et al., 1999 | Expanding the window for thrombolytic therapy in acute stroke. The potential role of acute MRI for patient selection. | Wrong study design: no direct comparison for CT and MRI-based reperfusion |
| Barber PA et al., 1999 | Identification of major ischemic change. Diffusion-weighted imaging versus computed tomography. | Wrong study design: no reperfusion therapy performed; lacks clinical comparison |
|  | CT or MRI in Work up for i.v. Thrombolysis: a Single-centre Study | Wrong study design |

**Supplementary Table 8. Characteristics of included studies.**

| **Author** | **Inclusion Criteria** | **Exclusion Criteria** |
| --- | --- | --- |
| **Fladt, J. et al, 2024** | ICA and/or M1 occlusion confirmed by CTA or MRA. Baseline NIHSS ≥5. t-PA within 4.5hrs of LSW. Thrombectomy within 75min of randomization | Significant premorbid disabilities. Advanced dementia. Baseline ASPECTS <5 in NCCT or DWI. |
| **Kamogawa, N. et al, 2022** | ≥20 years. ICA or M1 occlusion. LKW-to-hospital arrival <6hrs.  Pre-stroke mRS: 0-1. | ASPECTS for both NCCT and DWI. |
| **Krebs, S. et al, 2022** | NA | NA |
| **Li, J. et al, 2021** | ≥18years. t-PA within 4.5 window. | Unavailable angiographic imaging data. Unavailable mRS at 3 months |
| **Stosser, S. et al, 2021** | MRI or CT prior to ET. | Transferred patients with no pretreatment imaging. Baseline MRI and CT. Missing data on time of hospital admission. Absent Modality and time of neuroimaging. |
| **Atchaneeyasakul, K. et al, 2020** | Anterior circulation AIS-LVO. Baseline NIHSS ≥6. Thrombectomy. | NA |
| **Provost, C. et al, 2025** | 18-80 years. Proximal artery occlusion in CT or MRI. Baseline NIHHS 10-25. tPA within 4hrs of symptoms onset. Thrombectomy within 5hrs of symptoms onset. | NA |
| **Hansen, C. et al, 2017** | >17 years. Symptoms of AIS within 4.5hrs from onset. | Missing Visual Analog Scale questionnaires. |
| **Kang, D. et al, 2005** | Perfusion deficits without diffusion abnormalities. Any baseline NIHSS scores. t-PA within 3hrs of onset. | Increased risk for hemorrhagic complications. |
| **Solling, C. et al, 2009a** | 18-80 years. Presumed stroke. Acute infarct on DWI with or without PWI ⁄DWI mismatch. Baseline NIHSS <5. Seizures and mild strokes. | Hemorrhage or extensive infarction exceeding half of the MCA territory. |
| **Solling, C. et al, 2009b** | 18-80 years. NINDS criteria for stroke. Baseline NIHSS 1-25. Seizures and mild strokes. | MRI t-PA exclusion criteria: 1. Lesion appearing hyperintense on T2 Weighted image or FLAIR indicative of subacute infarction 2. ICH 3. Lesion size exceeding 1/3 of MCA and DWI/PWI mismatch 4. Lesion exceeding 1/2 of the MCA territory. CT exclusion similar to ECASS II criteria, any sign of hemorrhage and early ischemic changes exceeding /3 MCA |
| **Schellinger, P. et al, 2007** | Patients treated with standard dose t-PA. CT or MRI thrombolysis within 3h and MRI thrombolysis beyond 3hrs. | NA |
| **Kohrmann, M. et al, 2006** | Anterior circulation AIS. MRI or CT based t-PA <3hrs or MRI based t-PA >3hrs. | DWI lesion >50%. CT based t-PA >3hrs. |

***Supplementary Table 8 Abbreviations:*** ***AIS****, acute ischemic stroke;* ***LVO****, large vessel occlusion;* ***ECASS II****, European Cooperative Acute Stroke Study II;* ***ET****, endovascular treatment;* ***ICH****, intracerebral hemorrhage;* ***LKW****, last known well;* ***M1****, M1 segment of the middle cerebral artery;* ***NINDS****, National Institute of Neurological Disorders and Stroke*

**Supplementary Table 9: Baseline Clinical and Occlusion Characteristics of Included Studies**

| **Study** | **Baseline NIHSS score Case mean±SD** | **Baseline NIHSS score Control mean±SD** | **ICA occlusion Cases n(%)** | **ICA occlusion Control n(%)** | **M1 occlusion Cases n(%)** | **M1 occlusion Control n(%)** | **M2 occlusion Cases n(%)** | **M2 occlusion Control n(%)** | **IVT Cases n(%)** | **IVT Control n(%)** | **EVT Cases n(%)** | **EVT Control n(%)** |
| --- | --- | --- | --- | --- | --- | --- | --- | --- | --- | --- | --- | --- |
| Fladt J et al., 2024 | NA | NA | 52(26) | 62(30.2) | 136(68) | 131(63.9) | 12(6) | 10(4.9) | 105(52.5) | 100(48.8) | 200(100) | 205(100) |
| Kamogawa N et al., 2022 | 16±6.7 | 20.4±6.7 | 91(41.9) | 71(56.4) | 130(59.9) | 58(46) | NA | NA | 136(62.7) | 75(59.5) | 156(71.9) | 106(84.1) |
| Krebs S et al., 2022 | NA | NA | NA | NA | NA | NA | NA | NA | 2226 | 14573 | 373 | 1876 |
| Li J et al., 2021 | NA | NA | 70(40.7) | 124(42.7) | 95(55.2) | 148(51.0) | 27(15.7) | 52(17.9) | 172(100) | 290(100) | NA | NA |
| Stösser S et al., 2021 | NA | NA | 74(20) | 851(19.9) | NA | NA | NA | NA | 166(45.2) | 2157(50.7) | 360(100) | 4268(100) |
| Atchaneeyasakul K et al., 2020 | 16.4 ± 6.2 | 18.2 ± 6.3 | 16 (24) | 11 (28) | 37 (56) | 25 (62.5 ) | 13 (20) | 4 (10) | 30 (45) | 23 (58) | 66 (100) | 40 (100) |
| Provost C et al., 2019 | 18 ± 4.4 | 18 ± 4.4 | 45 (15) | 15 (15) | 252 (84) | 83 (83) | NA | NA | 152 | 51 | 147 (49) | 51 (50) |
| Hansen C et al., 2017 | NA | NA | NA | NA | NA | NA | NA | NA | 33 (24.6) | 107 (34.5) | NA | NA |
| Solling C et al., 2009 | NA | NA | NA | NA | NA | NA | NA | NA | 39(100) | 23(100) | NA | NA |
| Solling C et al., 2009 | overlap population with 22, 22 is more recent | overlap population with 22, 22 is more recent | overlap population with 22, 22 is more recent | overlap population with 22, 22 is more recent | overlap population with 22, 22 is more recent | overlap population with 22, 22 is more recent | overlap population with 22, 22 is more recent | overlap population with 22, 22 is more recent | overlap population with 22, 22 is more recent | overlap population with 22, 22 is more recent | overlap population with 22, 22 is more recent | overlap population with 22, 22 is more recent |
| Schellinger P et al., 2007 | NA | NA | NA | NA | NA | NA | NA | NA | 83 (100%) | 29 ( 100%) | NA | NA |
| Köhrmann M et al., 2006 | NA | NA | NA | NA | NA | NA | NA | NA | 496(100) | 714(100) | NA | NA |
| Kang DW et al., 2005 | 12.2±5.1 | 13±6 | NA | NA | NA | NA | NA | NA | 173(100) | 209(100) | NA | NA |

***Supplementary Table 9 Abbreviations:*** *EVT; Endovascular Therapy, ICA; Internal Carotid Artery, IVT; Intravenous Thrombolysis, M1; First Segment of the Middle Cerebral Artery, M2; Second Segment of the Middle Cerebral Artery, SD; Standard Deviation*

**Supplementary Table 10: Meta analysis Results MRI vs CT**

| **Outcome** | **Effect Size (95% CI)** | **Prediction Interval** | **SMD / MD / RR** | **I²** | **p-value** |
| --- | --- | --- | --- | --- | --- |
| Successful Reperfusion | 1.00 [0.91; 1.10] | [0.85; 1.18] | RR | 55.50% | 0.975 |
| 90-day mRS | −0.61 [−1.40; 0.18] | [−8.19; 6.97] | MD | 58.60% | 0.131 |
| mRS 1 (≤ 1) | 1.09 [0.94; 1.27] | [0.92; 1.29] | RR | 0% | 0.194 |
| mRS 2 (≤ 2) | 1.28 [1.06; 1.55] | [0.72; 2.29] | RR | 82.60% | 0.014 |
| Symptomatic ICH | 0.59 [0.45; 0.78] | [0.38; 0.93] | RR | 7.60% | 0.002 |
| Any ICH | 0.92 [0.77; 1.10] | [0.68; 1.24] | RR | 0% | 0.238 |
| Mortality | 0.72 [0.62; 0.84] | [0.59; 0.87] | RR | 0% | 0.002 |
| Hospital Mortality | 0.71 [0.55; 0.93] | [0.12; 4.20] | RR | 0% | 0.039 |

**Supplementary Table 11: Subgroup Analysis of Successful Reperfusion**

| **Successful Reperfusion** | **Category** | **k** | **RR** | **95% CI** | **I²** |
| --- | --- | --- | --- | --- | --- |
| **Risk of Bias (RoB)** | Low Risk | 3 | 1 | 0.75; 1.32 | 65.50% |
|  | Moderate Risk | 2 | 0.98 | 0.95; 1.01 | 0.00% |
|  | **Subgroup p = 0.78 (not significant)** |  |  |  |  |
| **Study Type** | RCT | 2 | 1.08 | 0.91; 1.28 | 0.00% |
|  | Cohort | 3 | 0.97 | 0.88; 1.07 | 0.00% |
|  | **Subgroup p = < 0.0001** |  |  |  |  |
| **Reperfusion Therapy Type** | EVT + IVT | 2 | 1.03 | 0.54; 1.96 | 83.00% |
|  | EVT | 2 | 0.89 | 0.46; 1.71 | 0.00% |
|  | IVT | 1 | 0.98 | 0.81; 1.20 | — |
|  | **Subgroup p = 0.13 (not significant)** |  |  |  |  |
| **CT Imaging Modality** | Multimodal CT | 4 | 1.02 | 0.93 to 1.12 | 50.1% |
|  | NCCT | 1 | 0.87 | 0.73 to 1.03 | -- |
|  | **Subgroup p = 0.08 (not significant)** |  |  |  |  |

**Supplementary Table 12: Subgroup Analysis of 90-day mRS**

| **90-day mRS** | **Category** | **k** | **MD** | **95% CI** | **I²** |
| --- | --- | --- | --- | --- | --- |
| **Risk of Bias (RoB)** | Low Risk | 1 | −0.19 | −0.95; 0.57 | — |
|  | Moderate Risk | 1 | −1.00 | −1.69; −0.31 | — |
|  | **Subgroup p = 0.12 (not significant)** |  |  |  |  |
| **Study Type** | Cohort | 1 | −0.19 | −0.95; 0.57 | — |
|  | RCT | 1 | −1.00 | −1.69; −0.31 | — |
|  | **Subgroup p = 0.12 (not significant)** |  |  |  |  |
| **Reperfusion Therapy Type** | IVT | 2 | -0.61 | -1.40 to 0.18 | 58.6% |
|  | EVT + IVT | 0 | _ | _ | _ |
|  | EVT | 0 | _ | _ | _ |
|  | **Subgroup p = ---** |  |  |  |  |
| **CT Imaging Modality** | Multimodal CT | 2 | -0.61 | -1.40 to 0.18 | 58.6% |
|  | NCCT | 0 | _ | _ | _ |
|  | **Subgroup p = ---** |  |  |  |  |

**Supplementary Table 13: Subgroup Analysis of mRS ≤ 2**

| **mRS ≤2** | **Category** | **k** | **Effect Size (RR / OR)** | **95% CI** | **I²** |
| --- | --- | --- | --- | --- | --- |
| **Risk of Bias (RoB)** | Low Risk | 3 | 1.16 | 1.12; 1.20 | 0.00% |
|  | High Risk | 1 | 1.2 | 1.11; 1.30 | — |
|  | Moderate Risk | 6 | 1.33 | 0.92; 1.94 | 91.00% |
|  | Moderate–High Risk | 1 | 1.7 | 0.98; 2.97 | — |
|  | **Subgroup p = 0.32 (not significant)** |  |  |  |  |
| **Study Type** | RCT | 3 | 1.22 | 1.02; 1.45 | 0.00% |
|  | Cohort | 8 | 1.31 | 0.99; 1.73 | 87.60% |
|  | **Subgroup p = 0.57 (not significant)** |  |  |  |  |
| **Reperfusion Therapy Type** | EVT + IVT | 3 | 1.26 | 1.15; 1.38 | 0.00% |
|  | EVT | 2 | 1.46 | 1.06; 2.02 | 0.00% |
|  | IVT | 6 | 1.9 | 1.05; 3.44 | 90.60% |
|  | **Subgroup p = 0.29 (not significant)** |  |  |  |  |
| **CT Imaging Modality** | Multimodal CT | 6 | 1.40 | 0.97 to 2.01 | 88.5% |
|  | NCCT | 5 | 1.13 | 0.10 to 1.29 | 42.4% |
|  | **Subgroup p = 0.16 (not significant)** |  |  |  |  |

**Supplementary Table 14: Influence Analysis of Successful Reperfusion**

| **Author (omitted)** | **Effect (95% CI: Lower; Upper)** | **I²** | **DFFITS** | **Cook’s D** | **QE (del)** | **Is Influential** |
| --- | --- | --- | --- | --- | --- | --- |
| Fladt J et al. 2024 | 0.97 (0.91; 1.03) | 0% | 1.64 | 0.839 | 1.68 | Yes |
| Kamogawa N et al. 2022 | 1.02 (0.93; 1.12) | 50.10% | −0.58 | 0.293 | 6.01 | No |
| Stosser S et al. 2021 | 1.01 (0.86; 1.19) | 48.90% | −0.17 | 0.042 | 5.87 | No |
| Atchaneeyasakul K et al. 2020 | 1.00 (0.87; 1.15) | 66.40% | 0.09 | 0.009 | 8.93 | No |
| Provost C et al. 2019 | 1.00 (0.87; 1.14) | 66.60% | 0.14 | 0.02 | 8.99 | No |

**Supplementary Table 15: Influence Analysis of 90 days mRS**

| **Author (omitted)** | **Effect (95% CI: Lower; Upper)** | | **I²** | **DFFITS** | **Cook’s D** | **QE (del)** | **Is Influential** |
| --- | --- | --- | --- | --- | --- | --- | --- |
| Atchaneeyasakul K et al. 2020 | −1.00 (−1.69; −0.31) | — | | 1.46 | 0.924 | 0 | Yes |
| Hansen C et al. 2017 | −0.19 (−0.95; 0.57) | — | | −1.67 | 1.082 | 0 | Yes |
|  |  |  |  |  |  |  |  |

**Supplementary Table 16: Influence Analysis of mRS ≤ 2**

| **Author (omitted)** | **Effect (95% CI: Lower; Upper)** | **I²** | **DFFITS** | **Cook’s D** | **QE (del)** | **Is Influential** |
| --- | --- | --- | --- | --- | --- | --- |
| Li J et al. 2021 | 1.15 (1.09; 1.22) | 0% | 2.86 | 1.588 | — | Yes |

 **Supplementary Table 17.** PRISMA 2020 Checklist

| **Section and Topic** | **Item #** | **Checklist item** | **Location where item is reported** |
| --- | --- | --- | --- |
| **TITLE** | | |  |
| Title | 1 | Identify the report as a systematic review. | Title Page:  Efficacy and Safety of Magnetic Resonance Imaging- versus Computed Tomography-Guided Reperfusion in Acute Ischemic Stroke: A Systematic Review and Meta-Analysis |
| **ABSTRACT** | | |  |
| Abstract | 2 | See the PRISMA 2020 for Abstracts checklist. | Abstract |
| **INTRODUCTION** | | |  |
| Rationale | 3 | Describe the rationale for the review in the context of existing knowledge. | Introduction paragraphs 1-3 |
| Objectives | 4 | Provide an explicit statement of the objective(s) or question(s) the review addresses. | Introduction – “Last paragraph” |
| **METHODS** | | |  |
| Eligibility criteria | 5 | Specify the inclusion and exclusion criteria for the review and how studies were grouped for the syntheses. | Methods – “Section 2.2 Eligibility criteria (2.2.1-2.2.4)” |
| Information sources | 6 | Specify all databases, registers, websites, organisations, reference lists and other sources searched or consulted to identify studies. Specify the date when each source was last searched or consulted. | Methods – “Section 2.3 Searching methods” |
| Search strategy | 7 | Present the full search strategies for all databases, registers and websites, including any filters and limits used. | Supplementary Tables 1–6 |
| Selection process | 8 | Specify the methods used to decide whether a study met the inclusion criteria of the review, including how many reviewers screened each record and each report retrieved, whether they worked independently, and if applicable, details of automation tools used in the process. | Methods – “Section 2.4 Selection of studies”  **Figure 1:** PRISMA flow diagram |
| Data collection process | 9 | Specify the methods used to collect data from reports, including how many reviewers collected data from each report, whether they worked independently, any processes for obtaining or confirming data from study investigators, and if applicable, details of automation tools used in the process. | Methods – “Section 2.5 Data extraction” |
| Data items | 10a | List and define all outcomes for which data were sought. Specify whether all results that were compatible with each outcome domain in each study were sought (e.g. for all measures, time points, analyses), and if not, the methods used to decide which results to collect. | Methods – “Section 2.5 Data extraction” |
|  | 10b | List and define all other variables for which data were sought (e.g. participant and intervention characteristics, funding sources). Describe any assumptions made about any missing or unclear information. | Methods – “Section 2.5 Data extraction” |
| Study risk of bias assessment | 11 | Specify the methods used to assess risk of bias in the included studies, including details of the tool(s) used, how many reviewers assessed each study and whether they worked independently, and if applicable, details of automation tools used in the process. | Methods – “Section 2.6 Assessment of risk of bias in included studies” |
| Effect measures | 12 | Specify for each outcome the effect measure(s) (e.g. risk ratio, mean difference) used in the synthesis or presentation of results. | Methods – “Section 2.7 Statistical analysis” |
| Synthesis methods | 13a | Describe the processes used to decide which studies were eligible for each synthesis (e.g. tabulating the study intervention characteristics and comparing against the planned groups for each synthesis (item #5)). | Methods – “Section 2.7 Statistical analysis” |
|  | 13b | Describe any methods required to prepare the data for presentation or synthesis, such as handling of missing summary statistics, or data conversions. | Methods – "Section 2.7 Statistical analysis" |
|  | 13c | Describe any methods used to tabulate or visually display results of individual studies and syntheses. | Methods – "Section 2.7 Statistical analysis" |
|  | 13d | Describe any methods used to synthesize results and provide a rationale for the choice(s). If meta-analysis was performed, describe the model(s), method(s) to identify the presence and extent of statistical heterogeneity, and software package(s) used. | Methods – "Section 2.7 Statistical analysis" |
|  | 13e | Describe any methods used to explore possible causes of heterogeneity among study results (e.g. subgroup analysis, meta-regression). | Methods – "Section 2.7 Statistical analysis" |
|  | 13f | Describe any sensitivity analyses conducted to assess robustness of the synthesized results. | Methods – "Section 2.7 Statistical analysis" |
| Reporting bias assessment | 14 | Describe any methods used to assess risk of bias due to missing results in a synthesis (arising from reporting biases). | Results – “3.3 Risk of Bias”  Results – “3.4 Meta-analysis”  Discussion “Paragraph 5”  “Supplementary Figures: 2” |
| Certainty assessment | 15 | Describe any methods used to assess certainty (or confidence) in the body of evidence for an outcome. |  |
| **RESULTS** | | |  |
| Study selection | 16a | Describe the results of the search and selection process, from the number of records identified in the search to the number of studies included in the review, ideally using a flow diagram. | Results – “Section 3.1 Study selection”  “Figure 1: PRISMA flow diagram” |
|  | 16b | Cite studies that might appear to meet the inclusion criteria, but which were excluded, and explain why they were excluded. | Results – “Section 3.1 Study selection”  “Supplementary Table 7 Reasons for Exclusion of Full-Text Articles” |
| Study characteristics | 17 | Cite each included study and present its characteristics. | “Table 1: Characteristics of included studies and general outcomes” |
| Risk of bias in studies | 18 | Present assessments of risk of bias for each included study. | Results – “Section 3.3 Risk of Bias Assessment”  “Figure 2 Risk of Bias Assessment for Included Randomized and non-Randomized Controlled Trials (RoB 2.0 and ROBINS-I Tool) (2A & 2B)” |
| Results of individual studies | 19 | For all outcomes, present, for each study: (a) summary statistics for each group (where appropriate) and (b) an effect estimate and its precision (e.g. confidence/credible interval), ideally using structured tables or plots. | Results – “Section 3.2 Individual study outcomes”  “Table 1: Characteristics of included studies and general outcomes” |
| Results of syntheses | 20a | For each synthesis, briefly summarise the characteristics and risk of bias among contributing studies. | Results – “Section 3.4 Meta-Analysis"  “Figure 2 Risk of Bias Assessment for Included Randomized and non-Randomized Controlled Trials (RoB 2.0 and ROBINS-I Tool) 2 (2A & 2B)” |
|  | 20b | Present results of all statistical syntheses conducted. If meta-analysis was done, present for each the summary estimate and its precision (e.g. confidence/credible interval) and measures of statistical heterogeneity. If comparing groups, describe the direction of the effect. | Results – “Section 3.4 Meta-Analysis"  “Figure 2 Risk of Bias Assessment for Included Randomized and non-Randomized Controlled Trials (RoB 2.0 and ROBINS-I Tool) 2 (2A & 2B)”  “Supplementary Figures 1 &2” |
|  | 20c | Present results of all investigations of possible causes of heterogeneity among study results. | Results – “Section 3.4 Meta-Analysis"  “Figure 2 Risk of Bias Assessment for Included Randomized and non-Randomized Controlled Trials (RoB 2.0 and ROBINS-I Tool) 2 (2A & 2B)” |
|  | 20d | Present results of all sensitivity analyses conducted to assess the robustness of the synthesized results. | Results – “Section 3.4 Meta-Analysis"  “Figure 2 Risk of Bias Assessment for Included Randomized and non-Randomized Controlled Trials (RoB 2.0 and ROBINS-I Tool) 2 (2A & 2B)” |
| Reporting biases | 21 | Present assessments of risk of bias due to missing results (arising from reporting biases) for each synthesis assessed. | “Supplementary Figure 2” |
| Certainty of evidence | 22 | Present assessments of certainty (or confidence) in the body of evidence for each outcome assessed. |  |
| **DISCUSSION** | | |  |
| Discussion | 23a | Provide a general interpretation of the results in the context of other evidence. | Discussion – “Paragraphs 1 & 2” |
|  | 23b | Discuss any limitations of the evidence included in the review. | “4.2 Limitations” |
|  | 23c | Discuss any limitations of the review processes used. | “4.2 Limitations” |
|  | 23d | Discuss implications of the results for practice, policy, and future research. | Discussion – “4.3 Future Recommendations” |
| **OTHER INFORMATION** | | |  |
| Registration and protocol | 24a | Provide registration information for the review, including register name and registration number, or state that the review was not registered. | Methods – “2.1 Methodology”  PROSPERO registered, ID: CRD420251165578 |
|  | 24b | Indicate where the review protocol can be accessed, or state that a protocol was not prepared. | The protocol can be accessed at: https://www.crd.york.ac.uk/PROSPERO/view/CRD420251165578 |
|  | 24c | Describe and explain any amendments to information provided at registration or in the protocol. | No amendments made. |
| Support | 25 | Describe sources of financial or non-financial support for the review, and the role of the funders or sponsors in the review. | “Statements” – “Funding” |
| Competing interests | 26 | Declare any competing interests of review authors. | “Conflict of interest” – None |
| Availability of data, code and other materials | 27 | Report which of the following are publicly available and where they can be found: template data collection forms; data extracted from included studies; data used for all analyses; analytic code; any other materials used in the review. | “Availability of data and materials” |
